# Poor-tasting pediatric medicines: part 2. Exploring caregiver and healthcare provider values and preferences for a novel taste-blocker product to improve acceptability

**DOI:** 10.3389/fddev.2025.1555522

**Published:** 2025-04-22

**Authors:** Moushira El-Sahn, Rose Elliott, Mona El-Sahn, Izaak Lucas, Karen Kong, Jennifer Walsh, Jeff Lucas

**Affiliations:** ^1^ Routes2Results, London, United Kingdom; ^2^ Jenny Walsh Consulting Ltd., Nottingham, United Kingdom

**Keywords:** drug formulation, drug delivery, palatability, africa, attributes, adherence, bitter

## Abstract

**Introduction:**

Improving the palatability of bitter-tasting medication for pediatric populations has long presented a challenge. Taste blockers are being researched as a potential solution; however, end-user perspectives and needs related to this concept have not been explored. The objectives of this research were 1) to understand current experiences of administering bitter-tasting medication; 2) the evaluation of a consumer-targeted product profile (CTPP) for a taste blocker including attributes such as form and duration of action; and 3) whether there is a need to support improved acceptability and adherence with a taste blocker taken before the bitter-tasting medication.

**Methods:**

Our study consisted of simultaneous qualitative and quantitative phases, involving caregivers and healthcare providers with experience administering medications to children aged 2–17 years. Qualitative research was conducted with 120 caregivers and 92 healthcare providers using a range of methods. Focus groups (FGs) were conducted in Kenya, Nigeria and Zimbabwe (grouped as Sub-Saharan Africa (SSA) but not intended to be representative of the region as a whole) with caregivers of children who had taken medication for HIV, TB, pneumonia, or malaria (including for seasonal prevention) within the past 6 months. Telephone in-depth interviews (TDIs) were conducted with caregivers of children with chronic illnesses in the United States. Face-to-face in-depth interviews (IDIs) and TDIs were conducted with healthcare providers. The quantitative part of the study was conducted with n = 1,815 caregivers and n = 859 healthcare providers using face-to-face computer-assisted interviews (CAPI) in SSA, and *via* online panel research in the United States A CTPP was used as the stimulus for discussion. Participants were asked about their experiences in giving bitter-tasting medication to their children or patients, their perceptions of and willingness to try a taste blocker, and their preferences for specific product attributes.

**Results:**

Participants described how bitter-tasting medications create challenges in multiple areas: for caregivers, children, their daily life and routines, healthcare providers, and children’s perceptions of healthcare. In SSA, 28.9% of caregivers reported that their children *always* or *regularly* refused medication due to bitter taste, while 57.9% reported this in the United States. Another 36.2% and 29.1% respectively experienced this *sometimes* or *occasionally*. Over 80% of providers in all countries stated that bitter taste impacts adherence to both long and short-term medication. The preferred attributes of the taste blocker were a sweetened and flavored lollipop form with a maximum total duration of up to approximately 1h, and with a total taste block achieved as soon as possible. Overall, responses to the concept of the taste blocker were positive from caregivers and providers, with a perception that it would make administering bitter-tasting medication easier. Over 90% were positive about using or prescribing the taste blocker in SSA, while in the United States, over 90% of caregivers were positive about using it, as were over 70% of providers about prescribing it. Concerns centered around the duration of the absence of the sense of taste, and the effects this might have on children’s appetite; there were also concerns that repeated taste blocking might have a long-term impact on children’s sense of taste.

**Conclusion:**

The results of the study indicate that there is a high perceived need for a taste blocker to aid in administering bitter-tasting pediatric medication. Concerns around duration and potential impact of long-term use must be addressed.

## Introduction

Although the burden of communicable diseases such as human immunodeficiency virus (HIV), malaria, and respiratory infections including tuberculosis (TB) has broadly declined over recent decades, there is still a high burden in low-middle-income-countries (LMICs), particularly in Sub-Saharan Africa (SSA) and South Asia. Moreover, the prevalence of communicable diseases, such as HIV, malaria, and TB, is disproportionately higher among children, particularly those under the age of five, compared to adults (Our World in Data, 2024). For example, pneumonia is reported to cause more deaths among children than any other infectious disease, causing 700,000 deaths of children under the age of five worldwide each year (UNICEF, 2024a), and an estimated 2.58 million children worldwide were living with HIV in 2022, with an estimated 100,000 AIDS-related deaths in children (UNICEF, 2024b). Globally, there were an estimated 263 million cases of malaria in 2023 and 597,000 deaths, with 94% of these cases (246 million) and 95% of these deaths (569,000) occurring in the WHO’s African region, of which children under 5 years accounted for approximately 76% of the deaths (World Health Organization, 2024b). The global TB incidence rate (new cases per 100,000 population per year) is estimated to have increased by 4.6% between 2020 and 2023, from 129 in 2020 to 134 in 2023, and in 2023 there were an estimated 1.3 million cases of TB among children and young adolescents (aged 0–14 years), equivalent to 12% of the estimated total (World Health Organization, 2024a). Despite the overall global rate of availability of essential medicines for children increasing slightly over recent years, the availability rate for systemic anti-infectives is reported to be low (Shi et al., 2023). The data above therefore underscores the critical need for increasing and ensuring consistent and equitable access to essential medications for children, particularly those under five, in LMICs.

It should be noted that in addition to maximizing access to medications to treat and prevent these diseases, it is important that medicines are age-appropriate and acceptable for the intended patient population. Patient acceptability is likely to impact patient medication adherence and is determined by the characteristics of both the product and user, with palatability being considered to be one of the main elements of patient acceptability of oral pediatric medicinal products (EMA, 2012). Indeed, it has been reported that poor taste is a common barrier to oral medicine administration in children, which may lead to lack of adherence and sub-optimal treatment outcomes (Lin et al., 2011; Mennella et al., 2015; Venables et al., 2015; Elgammal et al., 2023). For example, the poor taste of anti-retroviral medications such as ritonavir and nelfinavir has been reported to be a barrier to adherence, with a lack of adherence being associated with poor virologic response to therapy (Davies et al., 2008; Van Dyke et al., 2002).

Challenges associated with poor-tasing pediatric medicines are not limited to LMICs. For example, infectious diseases in children continue to be a major public health problem in the United States of America (United States) (Goto et al., 2016), and poor palatability, incomplete dosing and sub-optimal ease of use have been reported for some antibiotic and anti-pyretic/analgesic medications that are commonly used to treat such conditions (Cifaldi et al., 2004; Cohen et al., 2009; Klingmann et al., 2022; Smith et al., 2013). Furthermore, it has been estimated that approximately 25% of children and adolescents in the United States are affected by chronic conditions that require medication (Miller et al., 2016).

Many active pharmaceutical ingredients (APIs) have a bitter taste, and various taste-masking techniques have been developed and applied to formulations to improve their palatability (Ayenew et al., 2009; Kaushik and Dureja, 2014; Walsh et al., 2014; AL-Japairai et al., 2023; Hu et al., 2023). The perception of taste is through the interaction of molecules with taste receptors, and taste-masking strategies include the obscuration of taste *via* the addition of sweetening and/or flavoring agents, and the creation of a barrier between the API molecules and taste receptors, for example, by complexation or application of a coating (Ayenew et al., 2009; Kaushik and Dureja, 2014; Walsh et al., 2014; AL-Japairai et al., 2023; Hu et al., 2023).

Improving the palatability of APIs is likely to enhance patient acceptability and adherence, particularly in pediatric populations where taste remains a significant barrier to effective treatment (Baguley et al., 2012). Increased adherence to medications can directly translate into better health outcomes, reducing the disease burden and improving quality of life (Claxton et al., 2001; World Health Organization, 2003). Therefore, continued research and development into advanced taste-masking techniques and bitterness-blocking technologies are not only justified but essential for optimizing therapeutic efficacy and addressing global health challenges.

There has been increasing interest in the human taste pathway and the identification, development and use of compounds that can block the perception of bitterness at a molecular level, so called “bitter blockers”. Examples of commercially available and “generally regarded as safe” (GRAS) compounds that have been reported to show evidence of bitter blocking include sodium salts such as sodium acetate, sodium gluconate and sodium chloride, citric acid and adenosine 5′ monophosphate (Andrews et al., 2021). The perception of bitter taste is mediated *via* a family of around 25 G-protein coupled receptors (GPCR), the TAS2Rs. When a bitter compound interacts and binds with one or more TAS2Rs, this leads to the release of neurotransmitter that culminates in the activation of an afferent nerve fiber (usually the gustatory nerve) that transmits a signal to the brain (Mennella and Beauchamp, 2008; Mennella et al., 2013). Each TAS2R receptor is likely to recognize structurally similar compounds, and many APIs interact with multiple bitter receptors, leading to the need to apply more than one bitter blocker to fully block the taste. Indeed, the efficacy of a bitter blocker is compound specific and it has been reported that age may affect bitter blocking, potentially being less effective in children compared to adults (Mennella and Beauchamp, 2008; Mennella et al., 2014; Andrews et al., 2021; Flammer et al., 2024). Furthermore, there is some genetic variation in taste receptors which may lead to inter-subject variability in bitter blocking efficacy (Mennella et al., 2013; Nguyen et al., 2024). An alternative and emerging approach to blocking bitterness by antagonizing bitter taste receptors is *via* the prevention of the nerve signals that generate taste sensation from reaching the brain, although this would also affect other taste sensations such as sweet, sour, salt and umami (Flammer et al., 2024).

The blocking of bitterness taste perception using bitter or taste blockers may potentially be more effective compared to conventional methods of taste-masking, depending on the properties of the API and approach used. It may, therefore, offer a means by which the palatability and acceptability of a pediatric medicine can be improved, resulting in improved adherence and clinical outcomes in pediatric populations (Baguley et al., 2012; Claxton et al., 2001).

It is important to understand patient and caregiver experiences of administering and taking pediatric medicines and their perceptions of potential product development solutions to mitigate any challenges they face, to facilitate the development and administration of age-appropriate and acceptable pediatric medicines.

This research is complemented by an accompanying scoping review examining the impact of poor tasting pediatric medicines on patient acceptability, medication adherence, and treatment outcomes (Ranmal et al., 2024). The review highlights the global nature of the issue, which was found to affect children of all ages, with more than 150 unpalatable drugs identified across over 70 different disease areas. These findings underscore the need for more effective and universal taste-masking solutions such as a taste blocker.

## Objectives

There were three objectives of this research. Firstly, to understand current experiences of administering bitter-tasting medication. Secondly, to evaluate a sample CTPP for a taste blocker (blocking all taste perception, not specifically bitterness) and to determine caregiver and provider preferences around attributes such as form and time duration, and thirdly, to gather information on the need to support improved medication acceptability and adherence with a taste blocker. We conducted a multi-country qualitative and quantitative research study to gauge a broad range of stakeholder feedback and to gather information on caregivers’ experiences. The findings from this research will provide valuable insights to inform the work of funders and product developers in the field of pediatric drug development.

## Methods

### Overview

Our study consisted of qualitative and quantitative phases conducted simultaneously in Kenya, Nigeria, Zimbabwe and the United States. These countries were selected on the following basis: Kenya, Nigeria and Zimbabwe represent eastern, western and southern areas of Sub-Saharan Africa (SSA) and are low-middle socio-demographic index (SDI) countries, with high prevalence rates of four diseases linked with bitter-tasting medication; HIV, TB, pneumonia and malaria. In contrast, the United States is a high SDI country, offering a comparative context to explore the differences in experiences and challenges related to administering bitter-tasting medications. The sample comprised primary caregivers and pediatric healthcare providers (referred to as providers). For caregivers, qualitative research involved in-person focus groups (FGs) in Kenya (Nairobi), Nigeria (Lagos) and Zimbabwe (Harare) and telephone in-depth interviews (TDIs) in the United States (nationwide). The qualitative research with pediatric providers was conducted *via* face-to-face in-depth interviews (IDIs) in SSA and using TDIs in the United States.

The quantitative research was conducted using face-to-face (F2F) computer-assisted interviews (CAPI). Ethical approval was granted from in-country Institutional Review Boards (IRBs). Participants were primary caregivers and pediatric healthcare providers (referred to as providers). Those who took part in the qualitative research were not eligible to participate in the quantitative component. All interviews were conducted at a place of the respondent’s choosing, often in their home, or a space where they felt they could talk openly. The fieldwork was conducted between October 2023 and January 2024.

#### Qualitative phase: sample, data collection and analysis

FGs were conducted with caregivers in Kenya (Nairobi), Nigeria (Lagos) and Zimbabwe (Harare) and TDIs in the United States (nationwide). In SSA, FGs were conducted with 90 participants in total (n = 5 per group; n = 30 per country) and were 90–120 min in duration. IDIs in the United States were conducted with 30 participants and were 90–120 min in duration. The sample was divided evenly according to the ages of children cared for into three age groups: 2–5 years, 6–11 years and 12–17 years (n = 10 per age group in each country). Caregivers in Kenya and Nigeria were screened for inclusion based on whether they cared for children with chronic illness (HIV or TB) or children who had had pneumonia or malaria (treatment or seasonal malaria chemoprevention) in the 6 months preceding the study. In Zimbabwe, the caregiver sample was screened on whether they cared for children with HIV or TB only as malaria rates are low in this country. Participants in the United States were caregivers of children with chronic illness for which bitter-tasting medication is required daily over a long-term period. Qualitative research with pediatric providers was conducted *via* face-to-face in-depth interviews (IDIs) in SSA and TDIs in the United States. Providers in SSA were screened on prior experience in providing/administering medication to children with chronic illness (including TB, HIV, pneumonia and malaria); in the United States, providers were screened on their prior experience in providing/administering medication to children with chronic illness (unspecified). The breakdown can be seen in Table 1.

**TABLE 1:** Quantitative and qualitative sample.

Caregivers												
Qualitative sample: Caregivers												
Total (n=120)	n=30 per country (Focus Groups of n=5 in Kenya, Nigeria and Zimbabwe, individual telephone in-depth interviews in USA)											
	n=10 per country caregivers of children aged 2-5, n=10 caregivers of children aged 6-11, n=10 caregivers of children aged 12-17											
	• In Kenya, Nigeria and Zimbabwe, conditions of children cared for: HIV/TB, or pneumonia/malaria/use of SMC within past 6 months (50/50, except in Zimbabwe where all respondents cared for children with HIV/TB)											
	• In the USA, any condition for which bitter-tasting medication is taken daily, chronically											
Quantitative sample: Caregivers												
Total (n=1,815)	Kenya, n=401			Nigeria, n=407			Zimbabwe, n=406			USA, n=601 (panel)		
	2-5yrs	6-11yrs	12-17yrs	2-5yrs	6-11yrs	12-17yrs	2-5yrs	6-11yrs	12-17yrs	2-5yrs	6-11yrs	12-17yrs
	n=151	n=125	n=125	n=151	n=127	n=129	n=150	n=129	n=127	n=199	n=202	n=200
	• Distribution of conditions of children cared for in Kenya, Nigeria and Zimbabwe: HIV/TB (currently) 39%; pneumonia (within past 6 months) 22%; malaria or SMC (within past 6 months) 44% (some specified multiple conditions)											
	• In the USA, as for qual, there were no criteria for the condition apart from that bitter-tasting medication is taken daily, chronically											

Providers											
Qualitative sample: Providers (Face-to-face / telephone in-depth interviews)											
Total (n=92)	Kenya, Nigeria and Zimbabwe: total n=72						USA n=20				
	*Per country (n=24 total per country):*										
	Pediatricians		Pediatric Nurses		CHWs		Pediatricians			Pediatric Nurses	
	n=8		n=8		n=8		n=10			n=10	
Quantitative sample: Providers											
Total (n=859)	Kenya, Nigeria and Zimbabwe: total n=657						USA n=202 (panel)				
	Pediatricians	Pediatric Nurses		CHWs		Pharm.	Pediatricians	Pediatric Nurses	Retail pharmacists		Hospital pharmacists
	n=196	n=192		n=194		n=75	n=138	n=34	n=14		n=16

To analyze the qualitative data, codebooks were developed iteratively following review of transcripts by the core study team of four research directors, with a minimum of 5 years of qualitative analysis experience. This framework was used to code transcripts and identify key themes that emerged from the data. An iterative and systematic process of content and pattern analysis was carried out. The study team used the analytical categories developed as part of the coding framework to derive meaning from the various pieces of evidence to answer the research questions. The study analysis team met regularly to review codebook outputs, with a view to align/calibrate and/or resolve coding challenges; this included discussion and consensus-building, revisiting the codebook, and third-party review.

#### Quantitative phase: sample, data collection and analysis

The quantitative sample size was drawn on a stratified quota basis and a total 1,815 caregivers and 859 providers were interviewed (see Table 1). Data were collected *via* face-to-face (F2F) computer-assisted interviews (CAPI) in two cities per country in SSA (Kenya: Nairobi and Mombasa, Nigeria: Lagos and Abuja and Zimbabwe: Harare and Bulawayo). Interviews in the United States with both caregivers and providers were conducted *via* an online panel (AllGlobal). Quota sampling was used to allocate samples for each respondent category. Surveys lasted between 23 and 35 min for caregivers and between 26 and 36 min for providers, with the consent of the respondents granted where interviews overran (in the SSA countries). For data collection in SSA, mobile phones/tablets with offline data storage capability were used and data were automatically uploaded when an internet connection was available. All interviews were audio-recorded, transcribed, and, where necessary, translated prior to analysis. The research teams in SSA, comprising interviewers, recruiters and supervisors, were briefed and trained. Pilot interviews were observed across all countries, ensuring adherence to objectives, processes and ethical considerations. Interviews were conducted by experienced interviewers in the local language or English, based on respondent preference.

The closed-ended quantitative data were analyzed using International Business Machines (IBM)’s Statistical Package for the Social Sciences (SPSS). The dataset was cleaned and coded before analysis. Data were initially analyzed by total base size. Advanced analytical tools and approaches were then applied to make inferences about the target populations, with adjustments made to the data as needed. Statistical significance was determined using a P-value threshold of 0.05 (5%), and confidence intervals (CIs) were calculated at a 95% confidence level.

The tests used in the analysis included.• Z-test for the difference between two means:


$$\frac{X_{1}-X_{2}}{\sqrt{S^{2}\left(\frac{1}{e_{1}}-\frac{1}{e_{2}}\right)}}$$

Where.

X1 and X2 are the means of each group.

S2 is pooled variance.

e1 and e2 are the sample sizes of each group

• Z-test for the difference between two proportions:


$$\frac{p_{1}-p_{2}}{\sqrt{p_{p}\left(1-p_{p}\right)\left(\frac{1}{e_{1}}+\frac{1}{e_{2}}\right)}}$$

Where

p1 and 2 are the proportions of each group

pp is the pooled proportion

e1 and e2 are the sample sizes of each group.

#### Recruitment methods

Recruitment of caregivers (SSA): The recruitment process was consistent across all three countries. The target population was recruited using screening questionnaires (programmed and conducted on CAPI devices) which determined eligibility. Respondents were recruited from their households and healthcare facilities. This research utilized the EquityTool which is a short, country-specific questionnaire to measure relative wealth (Equity Tool, 2024). SEC strata C and D were selected for this research as they encompass the broadest and largest section of the population.

Recruitment of providers (SSA): a purposive sampling approach was used to recruit providers. Recruitment took place in-office in clinics and health facilities. All interviews were scheduled at a time which was convenient for the respondent to avoid disruptions to their regular schedule of activities, particularly as it pertains to their work. Providers were screened for eligibility (Table 1).

Recruitment of caregivers and providers in the USA: The data for this research study was collected through an online quantitative survey administered to participants drawn from a pre-existing online panel *via* AllGlobal. This approach leveraged the benefits of large, demographically diverse and readily accessible samples, ensuring that the sample was representative of the target population. Caregivers and providers were recruited *via* AllGlobal’s consumer/provider panels and the specific population needed for the study was targeted using screening questionnaires (programmed for quantitative and phone screening for qualitative) which determined eligibility.

#### Stimuli

**FIGURE 1:**
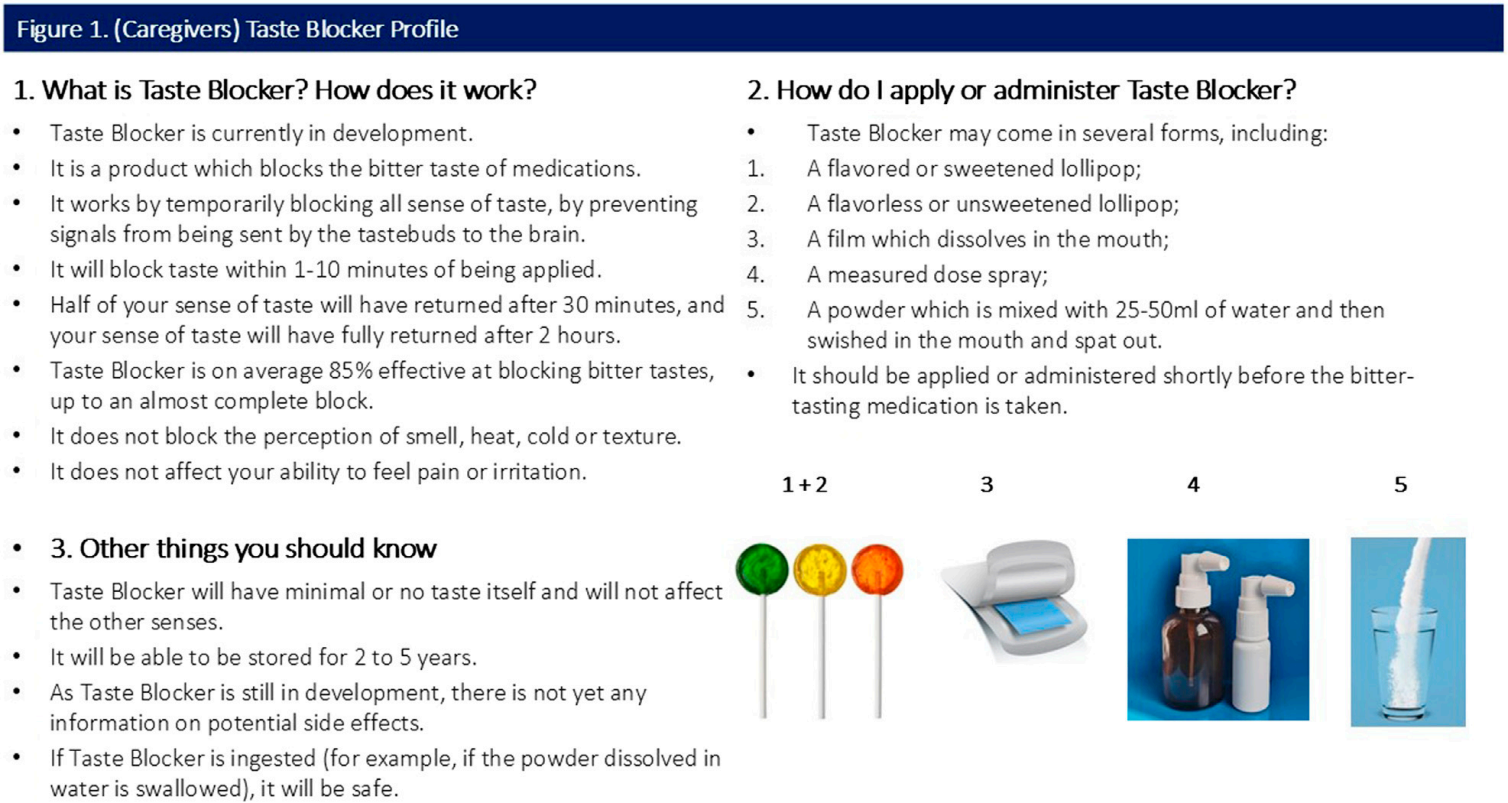
(Caregivers) Taste Blocker Profile.

**FIGURE 2:**
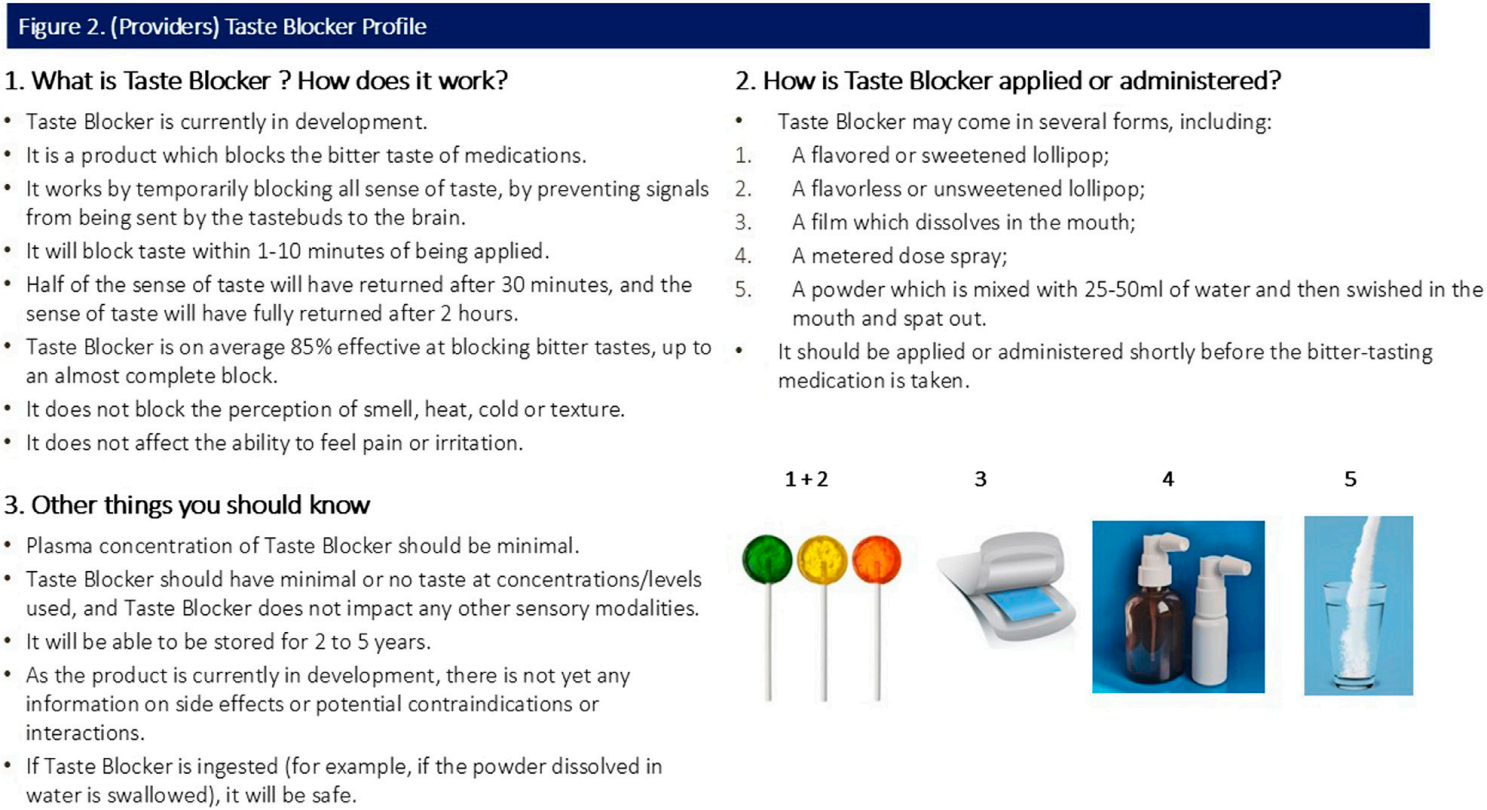
(Providers) Taste Blocker Profile.

All participants were shown a sample CTPP describing the function, usage and potential forms of the taste blocker. Caregiver and provider CTPPs are shown in Figures 1, 2 (please note, Section 3 of both figures contains specific information relevant to the caregiver and provider).

#### Translations

All informed consent forms, stimuli and research materials were translated into the main languages spoken in the areas where fieldwork was conducted: Kiswahili in Kenya, Pidgin in Nigeria, Shona and Ndebele in Zimbabwe. Respondents were able to choose languages for written materials and discussion, and to switch if preferred.

## Results

Presents the demographic information of the research participants.

The results presented in this paper represent the combined data from all four countries. Differences between individual SSA countries, as well as other data breakdowns, are present in the accompanying tables. Statistical significance is noted where a result from one country is significantly different from the other two (given as “significantly high” or “significantly low”). Statistically significant differences between caregivers of children of different age groups were also assessed; such differences are noted where salient (which was only in relation to forms of medication taken).

Where predefined lists of reasons were provided in the quantitative questionnaire an “Other–specify” option was also included. Results from the qualitative research are given to provide context to quantitative results.

### Objective 1: what is the experience currently?

#### Quantitative

Table 2 presents the 10 most cited dosage forms of bitter-tasting medication taken. Just under half (48.6%) of respondents in SSA overall described the form of the bitter-tasting medication as tablet - swallowed whole. Second and third most stated forms overall in SSA were liquid–on a spoon (24.5%) and tablet–crushed and put into water (21.3%). Kenya drove liquid–on spoon, with significantly more respondents selecting this form than Nigeria and Zimbabwe. Significantly more respondents in Zimbabwe selected tablet–dispersible in water (the second most selected form in Zimbabwe), and significantly more respondents in Nigeria selected tablet–break into pieces and swallow (joint second with liquid–on spoon).

**TABLE 2:** (Caregivers) Form of bitter-tasting medication taken (Top 10).

Caregivers	Total	Kenya	Nigeria	Zimbabwe	Caregivers	United States
Base	1,182	397	396	389	Base	601
Tablet - swallowed whole	48.6%	49.6%	52.0%	44.2%	Liquid - on spoon	28.5%
Liquid - on spoon	24.5%	35.8%^	24.7%	12.6%	Liquid - mixed with juice	28.0%
Tablet - crush and put in water	21.3%	23.7%	18.9%	21.3%	Liquid - mixed with water	27.5%
Tablet - dispersible in water	17.5%	8.8%	14.6%	29.3%^	Tablet - swallowed whole	26.8%
Tablet - break into pieces and then they are swallowed	14.4%	9.1%	23.5%^	10.5%	Liquid - syringed into mouth	23.6%
Tablet - crush and put in juice	7.7%	5.3%	9.1%	8.7%	Tablet - dispersible in water	22.1%
Liquid - mixed with water	6.6%	8.1%	8.8%	2.8%^a^	Tablet - crush and put in water	22.1%
Chewable tablet	5.6%	2.8%	7.8%	6.2%	Tablet - dissolves in mouth	22.0%
Liquid - syringed into mouth	5.6%	5.8%	3.8%	7.2%	Wafer - dissolves in mouth	20.0%
Tablet - crush and mix with food	4.7%	2.8%	6.3%	5.1%	Film - dissolves in mouth	19.5%

^a^ Significantly lower than the other 2 countries, ^ Significantly higher than the other 2 countries.

Overall, just under half of respondents from the United States panel sample stated they used liquid–on spoon (28.5%), liquid–mixed with juice (28.0%), liquid–mixed with water (27.5%) and tablet–swallowed whole (26.8%). There was a relatively even distribution across the remaining six forms of bitter-tasting medication, ranging between 23.6% and 19.5%.

In terms of significant differences between age groups, children aged 12–17 years were more likely to use the form tablet–swallowed whole in Kenya and Nigeria. Across the SSA countries, liquid forms (liquid–on spoon and tablet–crushed and put into water) were used significantly more among the youngest age group (2–5 years) (Table 2).

Table 3 shows the frequency with which children were reported to refuse medication due to its bitter taste across all countries. In SSA, 28.9% of caregivers reported this happened always or regularly, while a further 36.2% reported it happened sometimes (roughly once a month), or occasionally (Table 3). Just over a third of participants reported that they were not currently experiencing this problem. Significantly more respondents in Nigeria experienced refusals always or regularly (48.6%) compared with the other SSA countries, while in Zimbabwe, significantly more participants reported the problem occurred never or in the past (53%). In the United States, the percentage of caregivers reporting regular or frequent refusals was higher (57.9%), with only 13% of participants not experiencing the issue.

**TABLE 3:** (Caregivers) Frequency why child cared for had not taken medication due to the bitter taste.

Caregivers	Total	Kenya	Nigeria	Zimbabwe	Caregivers	United States
Base	1,214	401	407	406	Base	601
Yes (always, regularly)	28.9%	18.0%	48.6%^	19.9%	Yes (always, regularly)	57.9%
Yes, always (all or part of medication)	7.6%	4.5%	14.7%^	3.4%	Yes, always (all or part of medication)	29.6%
Yes, regularly (roughly once a week or more)	21.3%	13.5%	33.9%^	16.5%	Yes, regularly (roughly once a week or more)	28.3%
Sometimes or occasionally	36.2%	47.6%^	34.2%	27.1%^a^	Sometimes or occasionally	29.1%
Yes, sometimes (roughly once a month)	18.1%	21.2%	21.9%	11.3%^a^	Yes, sometimes (roughly once a month)	18.5%
It has happened occasionally or once	18.1%	26.4%^	12.3%	15.8%	It has happened occasionally or once	10.6%
No (never, happened in the past)	34.8%	34.4%	17.2%^a^	53.0.%^	No (never, happened in the past)	13.0%
It used to happen in the past, but no longer does	10.5%	7.0%	5.7%	19.0%^	It used to happen in the past, but no longer does	8.0%
No, never	24.3%	27.4%	11.5%^a^	34.0%^	No, never	5.0%

^a^ Significantly lower than the other 2 countries, ^ Significantly higher than the other 2 countries.

The vast majority of all providers across SSA and United States agreed that bitter taste impacts adherence to both long-term medication for chronic conditions (93.9% in SSA, 81.7% in United States) and to short-term medication for acute conditions (83.9% in SSA and 90.6% in United States) (Table 4). Similarly, most providers also stated that the bitter taste of medication regularly or sometimes caused problems when administered to children, both at an individual patient level (over 70% of respondents in both regions) and in a mass administration setting (around 90% of respondents in both regions; though the sample size in the United States was relatively small) (Table 5).

**TABLE 4:** (Providers) Extent to which bitter taste impacts adherence to medications in the long/short term.

PROVIDERS Extent to which bitter taste impacts adherence to long-term medication	Total	Kenya	Nigeria	Zimbabwe	PROVIDERS Extent to which bitter taste impacts adherence to long-term medication	United States
Base	657	202	244	211	Base	202
Yes	93.9%	96.0%	93.9%	91.9%	Yes	81.7%
No	5.2%	4.0%	4.1%	7.6%	No	9.4%
I do not know	0.9%	-	2.0%	0.5%	I do not know	8.9%

PROVIDERS Extent to which bitter taste impacts adherence to short-term medication for acute conditions	Total	Kenya	Nigeria	Zimbabwe	PROVIDERS Extent to which bitter taste impacts adherence to short-term medication for acute conditions	United States
Base	657	202	244	211	Base	202
Yes	83.9%	81.2%	87.7%	82.0%	Yes	90.6%
No	13.2%	17.3%	8.6%	14.7%	No	6.9%
I do not know	2.9%	1.5%	3.7%	3.3%	I do not know	2.5%

PROVIDERS Extent to which bitter taste of medications cause problems when administering to children, individually	Total	Kenya	Nigeria	Zimbabwe	PROVIDERS Extent to which bitter taste of medications cause problems when administering to children, individually	United States
Base	657	202	244	211	Base	202
Yes (regularly, sometimes)	77.0%	72.7%	93.0%^	62.5%§	Yes (regularly, sometimes)	70.3%
*Yes, regularly*	*36.8%*	*26.2%*	*54.1%^*	*27.0%*	*Yes, regularly*	*16.3%*
*Yes, sometimes*	*40.2%*	*46.5%*	*38.9%*	*35.5%*	*Yes, sometimes*	*54.0%*
Rarely / not a regular issue	13.3%	15.9%	3.2%§	22.2%^	Rarely / not a regular issue	24.2%
*It has happened, but is not a regular issue*	*9.3%*	*11.4%*	*2.0%§*	*15.6%*	*It has happened, but is not a regular issue*	*17.8%*
*It has happened, but only rarely*	*4.0%*	*4.5%*	*1.2%*	*6.6%*	*It has happened, but only rarely*	*6.4%*
Not a problem/do not administer medication to patients	9.8%	11.4%	3.6%§	15.1%^	Not a problem/do not administer medication to patients	5.5%
*No*	*5.5%*	*8.4%*	*2.0%§*	*6.6%*	*No*	*2.0%*
*I do not administer medication to patients*	*4.3%*	*3.0%*	*1.6%*	*8.5%^*	*I do not administer medication to patients*	*3.5%*

^§^ Significantly lower than the other two countries.

^ Significantly higher than the other two countries.

**TABLE 5:** (Providers) Extent to which bitter taste of medications cause problems when administering to children.

PROVIDERS In a mass administration setting	Total	Kenya	Nigeria	Zimbabwe	PROVIDERS In a mass administration setting	United States
Base	289	68	114	107	Base	20†
Yes (sometimes, regularly)	87.9%	83.8%	98.2%^b^	79.4%	Yes (sometimes, regularly)	90.0%
*Yes, regularly*	*42.9%*	*41.2%*	*53.5%*	*32.7%*	*Yes, regularly*	*25.0%*
*Yes, sometimes*	*45.0%*	*42.6%*	*44.7%*	*46.7%*	*Yes, sometimes*	*65.0%*
It has happened, but is not a regular issue	7.3%	5.9%	0.9%	15.0%	It has happened, but is not a regular issue	10.0%
No (rarely, no)	4.8%	10.3%	0.9%	5.6%	No (rarely, no)	-
*It has happened, but only rarely*	*1.4%*	*2.9%*	*0.9%*	*0.9%*	*It has happened, but only rarely*	*-*
*No*	*3.5%*	*7.4%*	*-*	*4.7%*	*No*	*-*

^a^ Significantly lower than the other 2 countries

^b^ Significantly higher than the other 2 countries.

#### Qualitative

The qualitative part of the study supported these findings, as bitter taste was the most commonly reported reason for difficulties in medicines administration from caregivers in all countries and across all age groups. Providers’ reasons for difficulty (Kenya, Nigeria and Zimbabwe only) were broader than those described by caregivers, with pill and dosage burdens a major concern as well as poor palatability.

“Almost every patient who comes in with chronic illnesses has worries about the administration of medication. They always have challenges, whether they are being taken in syrup or tablet form.”

Pediatric Doctor, Kenya

“…the taste is so bad that she says it stays in her mouth and she does not want to take it.”

Caregiver, 2–5 years, United States

### Objective 2: what would the taste blocker look like? what form would it ideally take?

#### Response to consumer-targeted product profile (CTPP)

Participants were shown a CTPP which briefly outlined the function, usage and attributes (including potential forms) of the taste blocker (Figures 1, 2). Overall quantitative reactions to the CTPP are outlined first, with the attributes then discussed in more detail with qualitative findings.

#### Preferred time durations

##### Quantitative

Overall, caregivers preferred the taste blocker to have a rapid onset of action (i.e., time to reach maximum effectiveness). In SSA, 40.3% of respondents preferred an immediate onset of action (increasing in Zimbabwe to 55.2%), or within 1–3 min (30.1%). In the United States, only 20% preferred an immediate onset and 29% of participants reported no preference for this time (Table 6). For providers, immediate onset was preferred (SSA: 39.4%; United States: 60.9%), followed by within 1–3 min (SSA: 30.9%; United States: 26.7%). Appeal of time to onset decreased from 4 min among caregivers and more strongly among providers.

**TABLE 6:** (Caregivers and Providers) Ideal time durations for Taste Blocker action.

CAREGIVERS Ideal time to reach maximum effectiveness	Total	Kenya	Nigeria	Zimbabwe	CAREGIVERS Ideal time to reach maximum effectiveness	United States
Base	1,214	401	407	406	Base	601
Immediate	40.3%	34.7%	31.0%	55.2%^	Immediate	20.0%
1–3 min	30.1%	29.7%	31.4%	29.3%	1–3 min	13.3%
4–6 min	16.2%	16.2%	23.6%^	8.9%^a^	4–6 min	14.0%
7–9 min	3.3%	3.0%	5.4%	1.5%	7–9 min	9.3%
10 min	10.0%	16.5%^	8.6%	5.2%	10 min	10.3%
Other	-	-	-	-	Other	4.2%
No preference	-	-	-	-	No preference	29.0%

CAREGIVERS Ideal length of time for taste masking	Total	Kenya	Nigeria	Zimbabwe	CAREGIVERS Ideal length of time for taste masking	United States
Base	1,214	401	407	406	Base	601
1–3 min	34.2%	35.7%	24.1%^a^	42.9%^	1–3 min	20.5%
4–6 min	28.2%	25.9%	31.7%	26.8%	4–6 min	12.0%
7–9 min	8.6%	5.7%^a^	10.3%	9.6%	7–9 min	11.1%
10–12 min	20.4%	22.2%	23.3%	15.8%^a^	10–12 min	10.0%
13–15 min	5.9%	6.2%	8.4%	3.2%^a^	13–15 min	7.8%
16–20 min	2.7%	4.2%	2.2%	1.7%	16–20 min	7.0%
Other	-	-	-	-	Other	2.8%
No preference	-	-	-	-	No preference	28.8%

CAREGIVERS Ideal time for half return of taste	Total	Kenya	Nigeria	Zimbabwe	CAREGIVERS Ideal time for half return of taste	United States
Base	1,214	401	407	406	Base	601
15–19 min	55.8%	50.9%	48.9%	67.5%^	15–19 min	22.3%
20–24 min	19.6%	18.2%	25.8%^	14.8%	20–24 min	9.7%
25–29 min	13.3%	18.7%	17.4%	3.9%^a^	25–29 min	9.7%
30–39 min	5.1%	7.2%	6.6%	1.5%^a^	30–39 min	6.3%
40–59 min	1.2%	2.0%	1.0%	0.7%	40–59 min	5.7%
1 h	0.4%	0.7%	0.0%	0.5%	1 h	5.3%
Over 1 h	0.2%	0.0%	0.0%	0.5%	Over 1 h	5.0%
Other	4.4%	2.2%	0.2%^a^	10.6%^	Other	3.5%
No preference	0.0%	0.0%	0.0%	0.0%	No preference	32.6%

CAREGIVERS Ideal time for full return of taste	Total	Kenya	Nigeria	Zimbabwe	CAREGIVERS Ideal time for full return of taste	United States
Base	1,214	401	407	406	Base	601
30–45 min	62.6%	52.4%^a^	69.5%	65.8%	30–45 min	24.1%
46–59 min	19.9%	19.2%	22.9%	17.5%	46–59 min	11.8%
60–89 min	7.1%	13.7%^	5.7%	2.0%^a^	60–89 min	7.2%
90–119 min	1.9%	4.2%^	0.7%	0.7%	90–119 min	7.0%
2 h	2.8%	8.0%^	0.0%	0.5%	2 h	4.8%
Over 2 h	0.2%	0.7%	0.0%	0.0%	Over 2 h	5.3%
Other	5.5%	1.7%	1.2%	13.5%^	Other	4.3%
No preference	0.0%	0.0%	0.0%	0.0%	No preference	35.4%

PROVIDERS Ideal time to reach maximum effectiveness	Total	Kenya	Nigeria	Zimbabwe	PROVIDERS Ideal time to reach maximum effectiveness	United States
Base	657	202	244	211	Base	202
Immediate	39.4%	41.6%	32.0%	46.0%	Immediate	60.9%
1–3 min	30.9%	24.8%	30.3%	37.4%	1–3 min	26.7%
4–6 min	15.8%	10.9%	27.5%^	7.1%	4–6 min	4.5%
7–9 min	3.7%	5.4%	3.3%	2.4%	7–9 min	2.0%
10 min	10.2%	17.3%^	7.0%	7.1%	10 min	1.0%
No preference	0.0%	0.0%	0.0%	0.0%	No preference	5.0%

PROVIDERS Ideal length of time for taste masking	Total	Kenya	Nigeria	Zimbabwe	PROVIDERS Ideal length of time for taste masking	United States
Base	657	202	244	211	Base	202
1–3 min	28.6%	35.6%	17.2%§	35.1%	1–3 min	31.7%
4–6 min	23.4%	17.3%	29.1%	22.7%	4–6 min	32.2%
7–9 min	10.0%	5.0%	15.6%	8.5%	7–9 min	10.4%
10–12 min	23.7%	26.7%	20.1%	25.1%	10–12 min	15.8%
13–15 min	9.1%	6.9%	13.5%	6.2%	13–15 min	4.5%
16–20 min	5.0%	8.4%	4.5%	2.4%	16–20 min	0.5%
No preference	0.0%	0.0%	0.0%	0.0%	No preference	5.0%

PROVIDERS Ideal time for half return of taste	Total	Kenya	Nigeria	Zimbabwe	PROVIDERS Ideal time for half return of taste	United States
Base	657	202	244	211	Base	202
15–20 min	58.8%	56.4%	45.9%	75.8%	15–20 min	67.8%
20–25 min	17.2%	9.9%	30.3%^	9.0%	20–25 min	9.9%
25–30 min	14.9%	20.8%	14.3%	10.0%	25–30 min	6.9%
30–40 min	6.2%	9.4%	7.4%	1.9%§	30–40 min	3.5%
40–60 min	1.4%	2.5%	0.8%	0.9%	40–60 min	1.0%
1 h	0.8%	0.5%	1.2%	0.5%	1 h	0.5%
Over 1 h	0.8%	0.5%	0.0%	1.9%	Over 1 h	0.5%
No preference	0.0%	0.0%	0.0%	0.0%	No preference	9.9%

PROVIDERS Ideal time for full return of taste	Total	Kenya	Nigeria	Zimbabwe	PROVIDERS Ideal time for full return of taste	United States
Base	657	202	244	211	Base	202
30–45 min	60.3%	52.0%	63.1%	64.9%	30–45 min	70.3%
45–60 min	15.5%	12.9%	20.9%	11.8%	45–60 min	11.9%
1 h to 1 and a half hours (60–90 min)	8.8%	9.4%	11.5%	5.2%	1 h to 1 and a half hours (60–90 min)	3.0%
1 and a half hours to 2 h (90–120 min)	3.5%	6.9%^	2.0%	1.9%	1 and a half hours to 2 h (90–120 min)	0.5%
2 h	5.2%	14.4%	1.2%	0.9%	2 h	0.0%
Over 2 h	0.2%	0.5%	0.0%	0.0%	Over 2 h	1.0%
Less than 30 min	6.5%	4.0%	1.2%	15.2%^	Less than 30 min	2.0%
No preference	0.0%	0.0%	0.0%	0.0%	No preference	11.4%

^a^ Significantly lower than the other 2 countries, ^ Significantly higher than the other 2 countries.

§Significantly lower than the other 2 countries, ^ Significantly higher than the other 2 countries.

Ideal durations for taste blocking effect reported by caregivers were up to 12 min, with most selecting 1–3 min (SSA: 34.2%, significantly high in Zimbabwe at 42.9% and significantly low in Nigeria at 24.1%; United States: 20.5%) and 4–6 min (SSA: 20.4%, significantly low in Zimbabwe at 15.8%; United States: 12.0%). The results for providers were similar: with 1–3 min (SSA: 28.6%, significantly lower in Nigeria at 17.2%; United States: 31.7%) and 4–6 min (SSA: 23.4%; United States: 32.2%) being the most selected options.

Both caregivers and providers strongly preferred that half of the normal sense of taste to return within 15–19 min (SSA caregivers: 55.8% and providers: 58.8%; United States caregivers: 22.3% and providers: 67.8%). The ideal time for the full return of the normal sense of taste was around 30–45 min, albeit less strongly expressed among caregivers in the United States (SSA caregivers: 62.6% and providers 60.3%; United States caregivers 24.1% and providers 70.3%).

**FIGURE 3:**
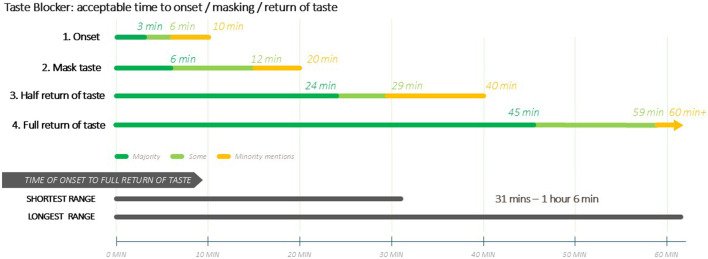
(Caregivers) Ideal time durations for Taste Blocker Action.

Figure 3 is a visual illustration of the acceptable time ranges for the taste blocker’s duration of action.

##### Qualitative

Caregivers and providers expressed that time for full taste to return should not take over an hour, nor 30 min for half return, and in some cases less, due to concerns over emotional and sensory discomfort (at not being able to fully taste food or drink or loss of appetite) and perceived potential harm. Longer lengths of taste blocker action were perceived to be impractical and therefore a barrier to use, especially if children were to take medicine multiple times a day. Caregivers were in general much more discriminating about time for the taste blocker to wear off as opposed to time for it to work. Others feared that long-term use might alter or damage the taste buds of the user, which could be of particular concern for children. Caregivers and providers expressed concern about the lack of information regarding side effects, drug-drug interactions, and potential impact on effectiveness of medication.

“Are we then going to be dealing with an unhappy child because they cannot taste anything for 2 hours, which will then bring more stress to the families as well? Is it just offsetting the stress to a different problem?”

Pediatric Doctor, United States

“The time is okay [30 min for half of normal sense of taste to return] because the child is supposed to take time before eating after taking medication. Also, the taste of medicine on the tongue would have disappeared as well.”

Caregiver, 6–11 years, Kenya

#### Extent of taste blockage

##### Quantitative

The majority of caregivers and providers overall (with the exception of providers in the United States who were more amenable to partial taste blocking) expressed a need for total taste blockage (Table 7) (caregivers: SSA 67.3%, United States 61.6%; providers: SSA 76.6%, United States 40.1%). Around one-third of the caregiver sample showed preference for partial blockage (SSA 31.7%, United States 34.0%); around a quarter among SSA providers (23.4%) and just over half of United States providers (54.9%).

**TABLE 7:** (Caregivers and providers) Extent of taste blockage.

Caregivers Extent of taste blockage	Total	Kenya	Nigeria	Zimbabwe	Caregivers Extent of taste blockage	United States
Base	1,168	397	390	381	Base	547
Total block required	67.3%	62.2%	63.1%	76.9%^	Total block required	61.6%
Partial block acceptable	31.7%	37.5%	34.9%	22.3%^a^	Partial block acceptable	34.0%
I do not know	1.0%	0.3%	2.1%	0.8%	I do not know	4.4%

Providers Extent of taste blockage	Total	Kenya	Nigeria	Zimbabwe	Providers Extent of taste blockage	United States
Base	282	66	114	104	Base	142
Total block required	76.6%	77.3%	73.2%	79.8%	Total block required	40.1%
Partial block acceptable	23.4%	22.7%	26.8%	20.2%	Partial block acceptable	54.9%
I do not know	0.0%	0.0%	0.0%	0.0%	I do not know	4.9%

^a^ Significantly lower than the other 2 countries, ^ Significantly higher than the other 2 countries.

#### Preferred forms

##### Quantitative

Overall, preference and acceptability of the form of the taste-blocker product were aligned across countries (Table 8), with a flavored/sweetened lollipop emerging as the most favorable option for caregivers and providers in each country. This product form was acceptable for over 70% of caregivers and providers in SSA and over 90% in the United States. This was followed by the spray and film, which had similar preference and acceptability scores. Caregivers and providers in Zimbabwe cited significantly greater acceptance of the spray and were also significantly more likely to prefer the film.

**TABLE 8:** (Caregivers and Providers) Preferred and acceptable Taste Blocker forms.

Caregivers Acceptable	Total	Kenya	Nigeria	Zimbabwe	Caregivers Acceptable	United States
Base	1,214	401	407	406	Base	601
A flavoured or sweetened lollipop	78.3%	75.6%	82.1%	77.1%	A flavoured or sweetened lollipop	90.5%
A film which dissolves in the mouth	40.8%	31.2%^a^	43.7%	47.3%	A film which dissolves in the mouth	56.6%
A measured dose spray	40.0%	30.2%	35.9%	53.9%^	A measured dose spray	52.6%
A powder which is mixed with 25–50 mL of water and then swished in the mouth and spat out	25.9%	27.4%	19.9%^a^	30.3%	A powder which is mixed with 25–50 mL of water and then swished in the mouth and spat out	48.3%
A flavourless or unsweetened lollipop	12.8%	6.7%^a^	15.7%	15.8%	A flavourless or unsweetened lollipop	48.3%

CAREGIVERS Preferred	Total	Kenya	Nigeria	Zimbabwe	CAREGIVERS Preferred	United States
Base	1,214	401	407	406	Base	601
A flavoured or sweetened lollipop	60.0%	60.6%	64.1%	55.2%	A flavoured or sweetened lollipop	66.6%
A film which dissolves in the mouth	15.1%	13.5%	10.8%	20.9%^	A film which dissolves in the mouth	24.4%
A measured dose spray	14.7%	13.5%	16.0%	14.5%	A measured dose spray	24.1%
A powder which is mixed with 25–50 mL of water and then swished in the mouth and spat out	7.4%	10.5%	4.2%^a^	7.6%	A powder which is mixed with 25–50 mL of water and then swished in the mouth and spat out	16.8%
A flavourless or unsweetened lollipop	2.9%	2.0%	4.9%^	1.7%	A flavourless or unsweetened lollipop	19.9%

Providers Acceptable	Total	Kenya	Nigeria	Zimbabwe	Providers Acceptable	United States
Base	657	202	244	211	Base	202
A flavoured or sweetened lollipop	76.4%	79.7%	77.0%	72.5%	A flavoured or sweetened lollipop	94.1%
A film which dissolves in the mouth	49.0%	42.1%	48.8%	55.9%	A film which dissolves in the mouth	77.7%
A measured dose spray	47.0%	36.1%	44.3%	60.7%^	A measured dose spray	74.3%
A powder which is mixed with 25–50 mL of water and then swished in the mouth and spat out	27.7%	22.8%	21.3%	39.8%	A powder which is mixed with 25–50 mL of water and then swished in the mouth and spat out	32.7%
A flavourless or unsweetened lollipop	17.7%	10.9%^a^	20.1%	21.3%	A flavourless or unsweetened lollipop	78.7%

PROVIDERS Preferred	Total	Kenya	Nigeria	Zimbabwe	PROVIDERS Preferred	United States
Base	657	202	244	211	Base	173
A flavoured or sweetened lollipop	53.9%	62.4%	52.9%	46.9%	A flavoured or sweetened lollipop	71.1%
A film which dissolves in the mouth	19.5%	15.3%	20.9%	21.8%	A film which dissolves in the mouth	19.1%
A measured dose spray	17.5%	14.4%	17.2%	20.9%	A measured dose spray	17.9%
A powder which is mixed with 25–50 mL of water and then swished in the mouth and spat out	6.8%	5.4%	6.1%	9.0%	A powder which is mixed with 25–50 mL of water and then swished in the mouth and spat out	2.9%
A flavourless or unsweetened lollipop	2.3%	2.5%	2.9%	1.4%	A flavourless or unsweetened lollipop	13.9%

^a^ Significantly lower than the other 2 countries, ^ Significantly higher than the other 2 countries.

Preference and acceptability continued to decrease with the powder followed by the flavorless/unsweetened lollipop with the exception of United States providers citing preference/acceptability of the flavorless/unsweetened lollipop ahead of the film. Looking at the SSA countries, caregivers in Kenya expressed significantly less acceptance of the film (SSA 40.8%, Kenya 31.2%) and to a lesser degree, the flavorless/unsweetened lollipop (SSA 12.8%, Kenya 6.7%).

While acceptability scores for all forms from both caregivers and providers were higher than preference scores (with respondents in the United States demonstrating higher acceptability scores compared to SSA), it is clear from the preference scores that the leading form of choice in all countries was the flavored/sweetened lollipop.

##### Qualitative

The qualitative findings below concentrate on the appeal of the flavored/sweetened lollipop which was found to be the most preferred form for a taste blocker product. While the form and delivery of the spray and film limited their overall appeal, these forms were evaluated comparably to one another. The powder, perceived as increasing workload and effort, was less well accepted, while the unflavored/unsweetened lollipop lacked the sensory draw (particularly for young children) and as such was seen as the least appealing option among all countries. Regardless of the product form, caregivers expressed confidence that the taste blocker would be safe if ingested and found a shelf life of 2–5 years to be both economical and practical.

Given that children are familiar with generic sweet lollipops, a flavored/sweetened lollipop was perceived as appealing and easy to use, acting as an incentive for (particularly younger) children to take medicine. These lollipops were perceived to be affordable and therefore favorable to incorporate into mass administration settings or to be used in-office when children visit their healthcare providers. Caregivers raised concerns regarding the potential for frequent sugar consumption and regarding potential misuse due to the lollipop’s appealing nature. Other concerns included whether an entire lollipop would be needed for each administration and, if not, whether the remaining portion could be reused or whether there would be wastage.

“I will start with the flavored sweetened lollipop. This is good, especially when with children. For example, if you give a child a sweet, they will like you, and this will put the patient at ease and [they] will quickly take the medication.”

Nurse, Zimbabwe

#### Sweetness and flavor preferences

##### Quantitative

###### Preference for flavor

The vast majority of caregivers and providers in SSA showed a preference for a flavored taste blocker product (Table 9). This was significantly high in Kenya (SSA 77.0%; Kenya 87.5%). Opinion was split between caregivers in the United States regarding flavor (yes, 39.1%; no, 41.3%), with around one in five caregivers citing no preference (19.6%). Having said this, providers in the United States tended towards preference for a flavored taste blocker (yes, 55.0%; no, 23.3%; no preference, 21.8%). Caregivers and providers in Zimbabwe were significantly more likely to report preference for no flavor. In Nigeria, caregivers and providers were significantly more likely to report having no preference.

**TABLE 9:** (Caregivers and Providers) Sweetness and flavor preferences.

Caregivers Preference for flavor	Total	Kenya	Nigeria	Zimbabwe	Caregivers Preference for flavor	United States
Base	1,214	401	407	406	Base	601
Yes, I would prefer it to have a flavor	77.0%	87.5%^	73.2%	70.4%	Yes, I would prefer it to have a flavor	39.1%
No, I would not prefer it to have a flavor	11.0%	7.2%	5.4%	20.2%^	No, I would not prefer it to have a flavor	41.3%
I have no preference	12.0%	5.2%^a^	21.4%^	9.4%	I have no preference	19.6%

CAREGIVERS Sweetened or unsweetened	Total	Kenya	Nigeria	Zimbabwe	CAREGIVERS Sweetened or unsweetened	United States
Base	1,214	401	407	406	Base	601
I would prefer flavorless and sweetened	83.4%	92.8%^	81.6%	76.1%	I would prefer flavorless and sweetened	73.5%
I would prefer flavorless and unsweetened	5.9%	4.5%	4.7%	8.6%^	I would prefer flavorless and unsweetened	14.3%
No preference	10.6%	2.7%^a^	13.8%	15.3%	No preference	12.1%

^a^ Significantly lower than the other 2 countries, ^ Significantly higher than the other 2 countries.

###### Preference for sweetness

The majority preferred the flavorless and sweetened option *versus* flavorless and unsweetened; this was again significantly high in Kenya. Also of note, caregivers in the United States more strongly opted for sweetness (73.5%, *versus* unsweetened at 14.3% or no preference at 12.1%) considering their split opinion on choice of flavor (Table 9).

###### Qualitative

Most caregivers and providers were in favor of having a flavored and sweetened taste blocker. Children’s familiarity with flavored medications was a contributing factor to flavor’s popularity. A number of respondents liked the idea of multiple flavor options to accommodate different preferences.

“No, I am back to square one if it did not have any taste. I am back to fighting again.”

Caregiver, 6–11 years, United States

“If it’s sweet they will take it, because all children love sweets. Sweet is one of the first taste buds that pop up.”

Pediatric Doctor, United States

### Objective 3: is there a need to support improved acceptability and adherence with a taste blocker taken before the bitter-tasting medication?

These findings focused on the respondents’ willingness to try or prescribe the taste blocker solution and examined the impact of bitter-tasting medications in three key areas: 1) the caregiver, child, and caregiver-child relationship; 2) daily life; and 3) providers, healthcare as a whole, and adherence. Despite the relatively low reported difficulty in administering bitter medicines using current strategies, there was a high perceived need for a taste blocker. Qualitative data were analyzed to help understand this discrepancy.

#### Current level of difficulty

##### Quantitative

Caregivers were asked to indicate, *via* a five-point Likert scale ranging from very easy to almost impossible, the level of difficulty they currently encounter when administering bitter-tasting medication, taking into account any strategies they use to make the process easier. Over half of caregivers indicated that it was very easy or easy to administer bitter-tasting medication using their current strategies (Table 10): in SSA, the overall proportion choosing these responses was 58.8% (50.6% in Kenya–significantly low – 59.7% in Nigeria and 66.0% in Zimbabwe). In the United States, it was 54.1%. These results correspond to the relative willingness to try scores in the four countries (see section willingness to try/prescribe).

**TABLE 10:** (Caregivers) Extent to which it is easy/difficult to administer medication with strategies.

Caregivers	Total	Kenya	Nigeria	Zimbabwe	Caregivers	United States
Base	657	202	244	211	Base	202
Very easy/Easy to administer	58.8%	50.6%^a^	59.7%	66.0%	Very easy/Easy to administer	54.1%
*Very easy to administer*	*11.4%*	*13.0%*	*10.6%*	*10.8%*	*Very easy to administer*	*20.5%*
*Easy to administer*	*47.4%*	*37.7%* ^a^	*49.1%*	*55.2%*	*Easy to administer*	*33.6%*
Slightly difficult	31.1%	36.4%^	28.7%	28.3%	Slightly difficult	27.1%
Very difficult/Almost impossible	10.0%	13.0%	11.5%	5.7%^a^	Very difficult/Almost impossible	18.8%
*Very difficult*	*9.6%*	*13.0%*	*10.1%*	*5.7%* ^a^	*Very difficult*	*13.1%*
*Almost impossible*	*0.5%*	*0.0%*	*1.5%^*	*0.0%*	*Almost impossible*	*5.7%*

^a^ Significantly lower than the other 2 countries, ^ Significantly higher than the other 2 countries.

There were a wide range of strategies being used which were primarily food- and drink-based, including to mask the taste–before, during or immediately after administration–or to wash away the taste using water or juice, or as a reward/inducement. Talking- and cooperation-based strategies were next most popular, including explaining to the child the importance of taking the medication, discussion/persuasion, and allowing the child to participate, wholly or in part, in administering the medication themselves.

#### Perceived need for taste blocker

##### Quantitative

Caregivers were asked to indicate their perception of the need for the taste blocker, using a five-point Likert scale ranging from yes, there is a definite need to no, there is no need at all. Perceived need was high overall (Table 11). Among caregivers in SSA, overall positive scores (definite need and somewhat of a need) were given by 93.2% of respondents (significantly higher in Kenya 98.0% and lower in Zimbabwe 86.2%). In the United States, positive scores were given by 76.5% of respondents.

**TABLE 11:** (Caregivers) Extent to which there is a need for a Taste Blocker.

Caregivers	Total	Kenya	Nigeria	Zimbabwe	Caregivers	United States
Base	1,214	401	407	406	Base	601
Yes	93.2%	98.0%^	95.3%	86.2%^a^	Yes	76.5%
*Yes, there is a definite need*	*69.0%*	*77.8%*	*84.8%^*	*44.6%* ^a^	*Yes, there is a definite need*	*35.1%*
*Yes, there is somewhat of a need*	*24.1%*	*20.2%*	*10.6%* ^a^	*41.6%^*	*Yes, there is somewhat of a need*	*41.4%*
I am not sure whether there is a need	6.1%	1.7%	3.7%	12.8%^	I am not sure whether there is a need	13.6%
No	0.7%	0.2%	1.0%	1.0%	No	9.8%
*No, there is not much of a need*	*0.6%*	*0.2%*	*1.0%*	*0.5%*	*No, there is not much of a need*	*7.2%*
*No, there is no need at all*	*0.2%*	*-*	*-*	*0.5%*	*No, there is no need at all*	*2.7%*

^a^ Significantly lower than the other 2 countries, ^ Significantly higher than the other 2 countries.

#### Willingness to try/prescribe

##### Quantitative

Caregivers and providers indicated a high level of willingness to try/recommend the taste blocker, with the highest levels in SSA. This was assessed *via* a five-point Likert scale, ranging from yes, I would definitely use/prescribe/recommend it to no, I would definitely not use/prescribe/recommend it (Tables 12, 13). In SSA, the overall caregiver result for the two most positive options (definitely and probably would use it) was 96.2% (99.0% in Kenya–significantly high – 95.8% in Nigeria and 93.9% in Zimbabwe). In the United States, the caregiver result for the two most positive options was 91.0%. For providers in SSA, the overall percentage selecting the two most positive options was 95.4%; in the United States it was 70.3%.

**TABLE 12:** (Caregivers) Likelihood to use Taste Blocker.

Caregivers	Total	Kenya	Nigeria	Zimbabwe	Caregivers	United States
Base	1,214	401	407	406	Base	601
Yes	96.2%	99%^	95.8%	93.8%	Yes	91.0%
*Yes, I would definitely use it*	*77.0%*	*86.3%*	*84.5%*	*60.3%* ^a^	*Yes, I would definitely use it*	*63.1%*
*I would probably use it*	*19.2%*	*12.7%*	*11.3%*	*33.5%^*	*I would probably use it*	*28.0%*
I am not sure whether I would use it	3.3%	0.7%^a^	3.7%	5.4%	I am not sure whether I would use it	7.2%
No	0.5%	0.2%	0.5%	0.7%	No	1.8%
*I would probably not use it*	*0.3%*	*0.2%*	*0.5%*	*0.2%*	*I would probably not use it*	*0.5%*
*No, I would definitely not use it*	*0.2%*	*-*	*-*	*0.5%*	*No, I would definitely not use it*	*1.3%*

^a^ Significantly lower than the other 2 countries, ^ Significantly higher than the other 2 countries.

**TABLE 13:** (Providers) Likelihood to recommend/prescribe Taste Blocker.

Providers	Total	Kenya	Nigeria	Zimbabwe	Providers	United States
Base	657	202	244	211	Base	202
Definitely/Probably would recommend/prescribe	95.4%	96.1%	94.7%	95.7%	Definitely/Probably would recommend/prescribe	70.3%
*Yes, I would definitely recommend/prescribe it*	*80.1%*	*83.2%*	*85.7%*	*70.6%* ^a^	*Yes, I would definitely use it*	*26.7%*
*I would probably recommend/prescribe it*	*15.4%*	*12.9%*	*9.0%*	*25.1%^*	*I would probably use it*	*43.6%*
I am not sure whether I would recommend/prescribe it	4.4%	3.5%	5.3%	4.3%	I am not sure whether I would use it	24.3%
Definitely/Probably would not recommend/prescribe	0.2%	0.5%	0.0%	0.0%	Definitely/Probably would not recommend/prescribe	5.4%
*I would probably not recommend/prescribe it*	*0.2%*	*0.5%*	*0.0%*	*0.0%*	*I would probably not use it*	*4.5%*
*No, I would definitely not recommend/prescribe it*	*0.0%*	*0.0%*	*0.0%*	*0.0%*	*No, I would definitely not use it*	*1.0%*

^a^ Significantly lower than the other 2 countries, ^ Significantly higher than the other 2 countries.

### Impact of taste blocker on caregiver, child, healthcare/healthcare provider dynamic

#### Qualitative

##### Impact on caregiver, child and caregiver-child relationships

Challenges impacting caregivers in all four countries related to three main aspects. The first of these was practical worries: whether the child is receiving enough doses and feeling responsible if they are not; being unsure how much has been spat out or vomited vs. consumed, and whether another dose ought to be given; for a few, the responsibility of being the one person who can give medicine. The second was negative emotional impact: the strain of having to give the child something they dislike *versus* wanting the child to get better or remain stable; the knowledge that this situation will be repeated over an ongoing period; that giving medication is a source of stress. The third aspect was concern around the strategies used, regardless of whether those strategies are successful (guilt around feeling the need to use force *versus* the risk of child not taking their necessary medication, or, for food-related strategies, concerns around sugar/confectionery intake). The taste blocker was perceived as having the potential to change these aspects positively, although downsides were also noted concerning cost, ongoing availability and the perceived potential harm of periods without the sense of taste.

In terms of impact on the child, the main issue cited by caregivers in all countries was fear of taking medication. This manifests in avoidance, and the outcome was described as children coming to see taking medication as a source of dread. The taste blocker was perceived as making the process of taking bitter-tasting medication potentially easier for children. Some caregivers noted that children would enjoy taking medication, either because of the inducement provided by the taste blocker, or simply because the experience of the bitter taste would be removed or reduced. Potential downsides relating to the child were around the creation of new stressors: possible reliance on taste blocker and refusal to take medication without it or attempts at accessing taste blocker outside the medication timetable.

Regarding the caregiver-child relationship, two main issues were identified by caregivers: those relating to the impact of using negative strategies (for example, arguing, telling off, use of physical force) and those relating to trust (both the caregiver’s trust in the child regarding taking the medicine correctly, and the child’s trust in the caregiver if pretending that the medication is not bitter is used as a strategy). The words “fight” and “battle” were used 25 times in context by caregivers, predominantly in the United States. The taste blocker was seen as potentially reducing conflict, and improving trust between parties regarding taking/administering medication.

##### Impact on daily life

Bitter-tasting medication was reported as causing a negative impact on day-to-day life due to time-consuming issues such as children’s unwillingness to take medication (including deliberately delaying taking it, or taking it very slowly), vomiting or spitting out doses (including potentially needing to change clothes), the need to devise strategies or distraction methods and the time taken to carry them out (for example, time spent watching cartoons or playing games as a prelude to medication). This issue was reported in all four countries, with the frequency of mentions in the following order: United States (numerous reports), Kenya, Nigeria, and Zimbabwe (very few reports). Some providers in the United States also noted being impacted by vomiting and by the time it takes to administer medication. The taste blocker was perceived by caregivers as potentially enabling time to be saved, as well as causing the daily routine to be less pressured (primarily mentioned in the United States); less need for caregiver supervision was also mentioned. In Kenya, Nigeria and the United States, saving time was highlighted as a reason for wanting to try the taste blocker or giving it a high “need” score. Some doctors in the United States stated that it could save time, both in terms of giving advice to caregivers and in their own practice.

##### Impact relating to providers, healthcare and adherence

Bitter-tasting medications can negatively impact children’s attitude towards and interactions with the healthcare system and providers–both because of the taste itself, and because of strategies used which foster a negative view. For example, caregivers using the threat of being spoken to by providers, going to hospital for treatment, or intravenous treatment to convince children to take the medication was seen to encourage a view of healthcare as something fear-inducing or negative. Children taking bitter-tasting medication chronically were reported by some providers to have come to associate medicine in general with bitterness.

In terms of impact on providers, some doctors in the United States reported calls and visits from caregivers facing challenges with giving medication, including out of hours. Resistance from caregivers to the prescription of certain medications was also brought up in discussion. The taste blocker was seen as potentially improving these aspects *via* improving children’s views of medicine and healthcare.

Furthermore, taste blocker was seen as potentially enabling in terms of which medications could be prescribed with greater confidence that they would be taken. Most doctors in SSA countries stated that they would change their prescribing decisions and would prescribe the most appropriate drug for the patient regardless of poor taste. In the United States, around half of doctors and almost all nurses stated that taste blocker would change their treatment decisions, on the grounds that they could prescribe drugs which are currently somewhat avoided because of taste (e.g., clindamycin and prednisone) despite high effectiveness. Reduced need to use intravenous administration as an alternative to oral medication was also noted as a potential positive (all countries). It was also seen as potentially positive regarding prescribing decisions around tablets, with providers in SSA reporting that tablets frequently need to be crushed or dissolved in water or food for children who have difficulty swallowing them, but that those which are not designed to dissolve may not have flavor-masking (and flavor-masking itself was described as not always successful). In all countries, around two-thirds of doctors stated that the taste blocker could impact whether liquids or tablets are prescribed.

With regards to adherence, caregivers reported frequent spitting out or vomiting post ingestion of medication, but there were divided views on whether second doses were given. Providers explained that missing doses causes issues with exacerbation of conditions, and caregiver fatigue leading to medication being stopped early. There was a strong perception among providers in all countries that the taste blocker could improve adherence to medication (for example, all doctors in Zimbabwe, half in Kenya and some in Nigeria mentioned this as a positive about the taste blocker after reading the profile).

## Discussion

This study has identified many valuable insights regarding stakeholder experiences of administering medicines to children, the strategies applied to overcome the challenges they experience, and their views on the properties, form and potential use of a taste blocker to facilitate medicines administration.

There was some variability between countries regarding bitter-tasting medication dosage form administered, for example, tablets, liquids, dispersible tablets and chewable tablets, which may reflect types of dosage form available and prescribed. It has been reported that monolithic dosage forms such as tablets and capsules are the dosage form of choice for adolescents (EMA, 2006), and tablets and capsules are generally perceived favorably by adolescents (Ranmal et al., 2016). Hence the observation in this study that adolescents were more likely to take tablets swallowed whole compared to younger children is not unexpected.

As stated above, poor taste has been reported to be a barrier to medicine administration to pediatric patients. Children dislike bitter taste, and compared to adults, they have a greater preference for sweet taste, which declines gradually throughout childhood, reaching adult levels in late adolescence (Mennella and Beauchamp, 2008). A recent scoping literature review investigating the impact of poor tasting pediatric medicines on patient acceptability, medication adherence, and treatment outcomes, conducted in parallel with this study (Ranmal et al., 2024), found that of the articles that reported poor taste as having an impact on patient acceptability, 24% included rejection, refusal or resisting the medication, with 13% reporting spitting out of the medicine. We also found that medicine refusal occurs and was reported to be always or regularly experienced by approximately 30% and 60% of respondents in SSA and United States, respectively. In addition, in the qualitative part of the study, bitter taste was the most commonly reported reason for difficulty in administering medicines.

Overall, the taste blocker concept had a positive response from both caregivers and healthcare providers regarding its perceived effect of facilitating bitter-medicine administration. The preferred and most accepted format was a sweetened and flavored lollipop, whilst a flavorless and unsweetened lollipop and powder mixed with 25–50 mL water and swished around the mouth and spat out were considered to be the least preferred and least acceptable formats. It is anticipated that familiarity with flavored and sweet lollipops as confectionary drove the preference and acceptance scores for this format, as well as them being perceived as easy to use and transport. However, there were concerns regarding potential sugar consumption. An unsweetened lollipop may be considered to be unpalatable to children, especially considering their preference for sweet taste (Mennella and Beauchamp, 2008). Types of flavour were not explored in the study, although a number of caregivers liked the idea of multiple flavor options. Children’s preferred flavors are likely to be driven by previous experience, culture and geographical location, and may also be influenced by gender. For example, a study in the United States reported that children’s favorite medicine flavors are cherry, bubble gum and grape (Mennella et al., 2015), whilst a survey conducted in India found that banana-vanilla, orange, lime, and orange-lemon flavours were preferred by males, and grape, banana-pineapple, and strawberry-lemon flavours were preferred by females (Panachiyil and Babu, 2021).

Providers in the United States particularly expressed a low preference for a powder for mixing with water, which then should be swirled in the mouth and spat out. This may have potentially been due in part to concerns regarding accidental swallowing, although this was not explored in the study. Ease of administration including complexity of dosage form modification prior to administration is a key attribute affecting patient acceptability (EMA, 2012). The need to dissolve a powder in a specified volume of water and get the child to swish the solution in their mouth and spit out before taking their medication may increase the time taken and burden associated with medicine administration for the caregiver or provider and the child, which may also have contributed to the negative perception of this format.

Onset of action of the taste blocker is an important attribute. Overall, both healthcare providers and caregivers preferred maximum effectiveness of the taste blocker to be reached immediately after administration, especially caregivers in Zimbabwe and providers in the United States. However, onset of action within a time period of 1–3 min was also accepted by many; this would enable medicine administration to swiftly follow the ingestion of the taste blocker.

The taste blocker concept presented to participants would result in the blockage of all taste, i.e., not only bitter taste. Therefore, the duration of taste blockage and time taken for taste to return were of key importance to caregivers, since a long duration of taste-blocking could interfere with other daily activities, especially eating, and could be a barrier to its use. There were variable results for ideal duration of taste masking, but this was broadly reported to be up to 12 min, although a minority of respondents accepted a duration of up to 20 min. This duration of action would be likely be sufficient to allow enough time for medication to be taken.

Variable results were also reported for ideal time for half of taste to return, although a time period of 15–19 min was most frequently reported by all groups. A preference for full taste to return within 30–45 min was expressed by the majority of participants, with some accepting a time period of up to an hour. It is anticipated that the preferred duration of taste blocking and time for taste to return reflect caregivers’ and providers’ perceptions of how a taste blocker may be effectively used without having a significant negative impact on daily routines, including mealtimes.

There was a high level of willingness to try or recommend the use of the taste blocker, and a high perceived need for a taste blocker, especially in SSA. However, it is interesting to note that over half of the caregivers reported that it was very easy or easy to administer bitter-tasing medication, when current strategies to facilitate this are taken into account. The use of food and/or beverage, before, with or immediately after medicine administration was widely reported. This is in line with the results of the scoping literature review which found that mixing medicine with food or beverage to help mitigate poor taste was reported in 24% of the studies identified (Ranmal et al., 2024), and the use of this approach to facilitate pediatric medicine administration has been reported elsewhere (Martir et al., 2020). Although this is a commonly used practice, there are concerns that it could result in aversion to the food or beverage within which the medicine is mixed, as well as having potential cost implications, especially in LMICs. In addition, unless assessed and indicated in the medicine product label, the impact of mixing a medicine with a food or beverage vehicle on product performance may not be known. For example, the vehicle may be incompatible with the medicine leading to potential API degradation or the disruption of protective pH sensitive coatings (FDA, 2018). In addition, the co-ingestion of food can affect the absorption and bioavailability of some APIs, although this will depend on type and quantity of food (Batchelor et al., 2018; Wiesner et al., 2023; Attia, 2024). What might be considered as being “negative” strategies to aid medicine administration were also reported, for example, the use of physical force and telling off. Similar strategies were reported in the scoping review (Ranmal et al., 2024): force, restraint, punishment and threats, as well as prescribers needing to change medication due to poor taste. The use of a taste blocker would clearly remove or reduce the need to apply some of the strategies described above to aid the administration of bitter-tasting medicines. Indeed, the potential risks associated with food/beverage-medicine interactions would be mitigated, stress on both the child and caregiver would be reduced, and time taken for medicine administration would be shortened.

We are not aware of any other published studies on healthcare provider and caregiver perceptions and preferred properties of taste blockers. As discussed above, the perception of bitter taste may be blocked by molecules that either antagonize bitter receptors or prevent the transmission of nerve signals to the brain that generate taste sensation. Recent research has been investigating both approaches to help mask the taste of various APIs used for the treatment or prevention of various LMIC prevalent diseases. For example, it has been found that the antiretroviral (ARV) drug tenofovir alafenamide (TAF) is predominantly activated by the taste receptors TAS2R39 and TAS2R21, as well as others, and pre-rinsing with the bitter blocker 6-methylflavone resulted in the blocking of bitterness in some subjects, although there was a difference in blocking efficacy between different subjects (Schwiebert et al., 2021). Pre-rinsing or mixing drug with thiazolidinediones has been found to partially block the bitterness of TAF and praziquantel, a drug used to treat schistosomiasis (Nguyen et al., 2024). Blockers of P2X2/P2X3 receptors act by preventing the activation of taste nerves, thereby preventing the transmission of nerve signaling to the brain. A study by Flammer et al. (2024) found that the topical oral application of a P2X2/P2X3 blocker transiently suppressed the bitter taste of quinine hydrochloride, praziquantel, sucrose octaacetate, TAF and urea, as well as suppressing salt, sweet, sour and savoury taste sensations. No effects were seen on other oral or nasal sensations such as astringency.

It is anticipated that the development and use of a transient “universal” taste blocker, as investigated by Flammer et al. may be more appropriate than blockers targeting specific bitter taste receptors, due to their potential greater utility. Indeed, unlike molecules that only block specific bitter taste receptors (“bitter blockers”), a single universal taste blocker may be effective at blocking the unpleasant taste of a wide range of APIs.

Based on the findings of this study and recent bitter and taste blocker research, it is proposed that a universal taste blocker would be of benefit to patients and their caregivers, to facilitate the administration of bitter tasting medicines to children. Indeed, the application of a universal taste blocker shortly before medicine administration will reduce and potentially eliminate the need for caregivers and providers to use strategies such as mixing the medicine with food or beverage, coercion, restraint or force, to ensure the medicine is taken. This will reduce the burden of taking unpleasant tasting medicines on children, caregivers and providers, and mitigate the negative impact this can have on daily life, and relationships with family members and providers. Furthermore, the risk of the child vomiting, refusing or spitting out the bitter-tasting medicine will be reduced, thereby improving adherence and clinical outcomes.

## Limitations

A significant limitation of this study is that caregivers and providers provided their views on children’s experiences and perceived opinions and preferences, but not children themselves. Clinical studies would likely include children if indicated for pediatric use. Furthermore, concepts such as “half of normal taste” were explored perceptually rather than empirically, and so evaluation depends on individual participant interpretation. Future clinical research should also examine any drug-drug interactions for priority medications that are bitter and would be most likely used with a taste blocker.

We do not recommend drawing broad conclusions across and between different country populations. The qualitative portion of the study is indicative in nature, and further, qualitative samples often fluctuate as not all questions are asked or answered. The scope of our research did not include development of data for a segmentation, a forecast or modeling, product pricing or price sensitivity evaluation.

## Conclusion

Our study demonstrates that there is a need for a taste blocker designed for administration immediately prior to bitter-tasting pediatric medication to support adherence, because of widespread difficulties currently experienced. There is a strong preference for a flavored/sweetened lollipop form and general preference for a limited duration of action. Concerns around side effects, drug-drug interactions and perceived potential long-term impacts on the developing sense of taste must be addressed. Overall, there is a positive response to the taste blocker concept from caregivers and providers.

## Data Availability

The raw data supporting the conclusions of this article will be made available by the authors, without undue reservation.
